# Regionalised Life Cycle Assessment of Bio-Based Materials in Construction; the Case of Hemp Shiv Treated with Sol-Gel Coatings

**DOI:** 10.3390/ma12182987

**Published:** 2019-09-15

**Authors:** Mohammad Davoud Heidari, Michael Lawrence, Pierre Blanchet, Ben Amor

**Affiliations:** 1 Interdisciplinary Research Laboratory on Sustainable Engineering and Eco-Design (LIRIDE), Faculty of Engineering, Department of Civil and Building Engineering, Université de Sherbrooke, 2500 boul. de l’Université, Sherbrooke, QC J1K 2R1, Canada; 2 NSERC Industrial Research Chair on Ecoresponsible Wood Construction (CIRCERB), Forest and Wood Sciences Department, Université Laval, 2425 rue de la Terrasse, Quebec City, QC G1V 0A6, Canada; 3 BRE Centre in Innovative Construction Materials (BRE CICM), Department of Architecture and Civil Engineering, University of Bath, Bath BA2 7AY, UK

**Keywords:** bio-based material, building envelope, hempcrete wall, hemp shiv, regionalized LCA, carbon sequestration

## Abstract

Interest in intrinsically low-energy construction materials is becoming mainstream, and bio-based materials form a key part of that group of materials. The goal of this study was to analyse the environmental impact of applying a sol-gel coating on hemp shiv, in order to improve the durability of this innovative bio-based material, using a regionalised LCA model, taking into account regional specific peculiarities. This study analysed the environmental performance of using bio-based materials in the building envelope compared with traditional synthetic construction materials, and compared the impact of a regionalised approach with a global approach. The carbon footprint of treated hemp shiv in a wall with a U-value of 0.15 W/m^2^.K was compared to untreated hempcrete and a reference cavity wall with the same U-value. Considering the environmental damage caused by the production of hemp shiv, nitrogen fertiliser was the hotspot. The LCA results showed that, using innovative bio-based materials in construction, treated hemp shiv with sol-gel can decrease the carbon footprint of a building envelope through carbon sequestration. Using the more accurate site-specific information in life cycle inventory and impact assessment methods will result in more consistent and site-appropriate environmental results for decision-making.

## 1. Introduction

The construction sector has a vast environmental impact and, specifically, this sector is responsible for producing a huge amount of CO_2_ emissions, for example, the building sector accounts for around 40% of the total emissions in the UK [1]. Choosing materials with a high impact on the environment and energy consumption would increase the total environmental loads of a building.

Whereas in the past the construction sector primarily used materials that were local to the construction site (with low environmental impact and energy consumption), nowadays this sector uses global materials (e.g., concrete, gypsum, steel, aluminium and polyvinyl chloride (PVC)) with high environmental impact and energy consumption. Innovative bio-based materials (materials produced from biomass) such as hemp shiv are being developed for use in construction, with the goal of being more environmentally friendly during their life cycle.

Hemp as a variety of *Cannabis sativa* L. is an annual plant and can be grown in a range of climates. Hemp has a woody core that is called shiv. Due to notable thermal resistance, and mechanical and acoustic features, hemp shiv has been recently used a bio-based material in the building industry (e.g., paper, lightweight composites). Since hemp shiv has a highly porous structure, in the very first minutes of exposure it can absorb a large amount of water [2]. This hydrophilic nature of hemp shiv makes it potentially a less durable material because of the risk of colonial fungal growth.

Bio-based coatings are promising materials to prevent building materials from fungal, damp and keep their durability [3]. A number of coating materials have been reported to reduce water absorption of different building materials such as wood [4,5,6] and natural fibres [7,8,9,10]. Considering the water resistance, coating materials are classified as hydrophilic and hydrophobic coatings. Functionalised sol-gel as a hydrophobic coating material is used to modify hydrophilicity of hemp shiv and also to reduce its water absorption rate and to enhance its water resistance [11]. Hemp cultivation was common in Europe up to the 1980s, but then went into a decline for a decade until its potential for insulation and industrial fibres was recognised. France, as the largest producer of hemp in Europe, uses 12,000 t of hemp yearly in the construction industry as an insulation building material. The use of hemp is growing across Europe and hundreds of buildings use hemp products as an environmentally-friendly material to fill roofs, walls and also under floors [12]. 

Even though the introduction of innovative bio-based material will reduce the carbon footprint compared with fossil-based materials [13], it may incur some additional costs (land use and its related environmental impacts). Environmental assessment is required to make a strategic decision related to the use of these bio-based materials instead of their fossil-based ones.

Life cycle assessment (LCA), as one of the most powerful approaches, is used to analyse the environmental impacts of a range of products and services throughout their life cycle (i.e., from raw material extraction, manufacturing, use phase through to end-of-life stage) [14]. LCA in the design stage of construction projects is used to analyse the materials and energies either for the goal of comparing different scenarios or applying improvements. Some environmental impacts (e.g., water use and photochemical ozone formation) have site-specific characterisation factors (CF)s, and they vary depending on their geographical characteristics and; therefore, on the location of the activity. Regionalised LCA is a technique that takes into account this variability [15].

Previous LCAs has been conducted on a wide range of bio-based materials, including hemp-concrete (hempcrete) [16,17,18], fibre composites [19], starch-based polymers [20] and bio-based plastics [21]; however, regionalised LCA of bio-based materials has received less attention. 

A review of the literature shows that climate change is the main indicator that has been considered to analyse the environmental impact of hemp shiv as an aggregate for insulation walls [22]. This study will consider other environmental impacts of a hempcrete wall using a different impact assessment method. Based on the authors’ best of knowledge, there is no study on the environmental impact of treated hemp in a building envelope. The other key novelty of this study is the consideration of site-specific characterisation factors in order to assess the environmental impacts of hemp shiv production.

Toward this end, the goal of this study was to analyse the damage to terrestrial biodiversity and carbon footprint from the production of hemp shiv treated with sol-gel-based materials per functional unit (FU) of 1 kg of hemp shiv produced. Treated hemp shiv as a bio-based composite wall with thermal transmittance (U-value) of 0.15 W/m^2^.K was compared to two other walls with the same U-value, which were a wall made with untreated hemp shiv and a reference cavity wall constructed using traditional masonry techniques. Damage to the terrestrial ecosystem due to land use, acidification, photochemical ozone formation, ecotoxicity, water use and global warming was included.

## 2. Materials and Methods

The studied product and its production process are described in detail in the following paragraphs, followed by an explanation of the regionalised LCA modelling applied.

International Organization for Standardization (ISO) standards 14040 and 14044 have defined a comprehensive procedure for conducting an LCA [14]. There are four main steps, which starts with goals and scope definition. The main goal of the study is established in this step, following by defining geographic and temporal boundaries of the study. The FU (i.e., a reference that the inputs and outputs are quantified based on that) is defined in this step as well. The second step of an LCA study is a life cycle inventory (LCI), where data for all processes are collected according to the FU. Life cycle impact assessment (LCIA) is the following step, where all collected data (including inventories from natural resource and emissions to air, soil, and water) are converted to environmental impact assessment midpoint and endpoint categories, using related characterisation factors. Each of calculated impact categories at this step addresses a specific environmental concern. The results of the environmental impacts of an LCA study are interpreted at the final step, which is referred to as interpretation [23].

### 2.1. Goal and Scope

**Figure 1:**
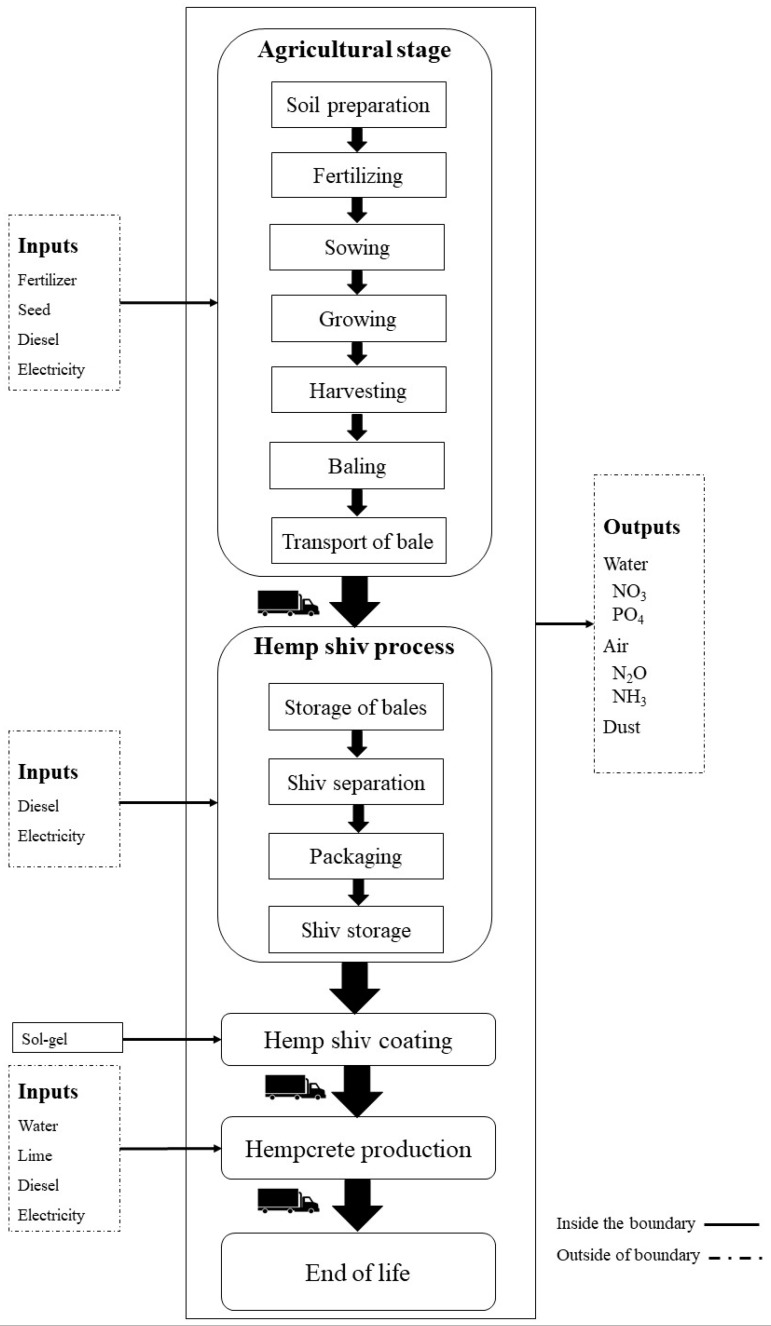
Life cycle and system boundaries of the hempcrete production.

Data on hemp production were obtained from hemp farmers from CAVAC, an agricultural cooperative based in west France. Potentially disappeared fraction of species (PDF), as a damage to the terrestrial biodiversity, integrated over area and time in m^2^ y, was analysed for the functional unit of 1 kg of hemp shiv and sol-gel coating material produced, and subsequently per one square meter of insulation wall with a U-value of 0.15 W/m^2^.K for one year of its service life. The system boundary for hemp shiv production, as it is shown in Figure 1, include all processes from agricultural soil preparation to hemp harvesting, its transportation to the processing plant and shiv storage. The scope of the study for insulation walls includes the hempcrete production and its end-of-life stage, as well. Agricultural stage (production of hemp plant) and hemp shiv processing (shiv production) (Figure 1).

### 2.2. Life Cycle Inventory

#### 2.2.1. Hemp Production

Hemp was produced in the western part of France (Vendée) and was collected from local farmers. Western (Le Mans) and northeastern (Troyes) regions of France are the main producers of hemp plant [24]. The hemp shiv production has two stages. The agricultural stage of hemp production begins with soil preparation. This agricultural operation is used to create a crumbled seedbed that retains sufficient surface moisture to facilitate germination. It is generally carried out in two stages: An early winter ploughing to allow the action of frost followed by a recovery in early spring ploughing and preparation of the seedbed.

The necessary elements to produce hemp are nitrogen (N), phosphate (P_2_O_5_) and potassium (K_2_O). Depending on the soil condition, the amount of nitrogen fertiliser varies between 80 and 120 kg/ha. Nitrogen is often used in the form of ammonium nitrate during the spring recovery [25]. Phosphate and potassium are often applied during the winter ploughing. Sowing is done around mid-April in France. Sowing densities range from 40 to 70 kg/ha and its depth varies between 2 and 3 cm. Harvesting of hemp takes place when the seeds are ripe. In France, hemp harvesting is usually started after mid-September. A conventional harvester is used to cut the hemp and once the hemp seed is harvested, the hemp straw is mowed and collected. The hemp straw is mowed using a mower conditioner. They are then left in the field to dry and ret. The straw yield of hemp is between 8 and 12 t/ha [24].

After the farmers establish that the straw is dry, the straw is transported to a primary processing site. On this site, hemp straw is mechanically treated to separate the cortical fibre (from the bark of hemp) and the hemp seed (from the marrow of the hemp stalk). Hemp dust (also called powder) is waste resulting from this separation. Since this product (dust) has economic value, it was considered as by-product in this study. The fibre and the shiv are then transported to the packaging sites. Hemp shiv processing required 79 kWh electricity, 4.82 L diesel and 0.3 kg propane for each tone of stalk [18]. The inventory data of the hemp crop and hemp shiv production are shown in Table 1.

#### 2.2.2. Emissions to Air and Water

The Ecoinvent 3.4 database was applied to calculate the emissions from fertiliser production [26]. The emissions of nitrate, phosphate, nitrous oxide and ammonia associated with application and leaching of the fertiliser were calculated.

Nitrate (NO_3_^−^) emission is 40 kg/ha by leaching. Phosphate (PO_4_^3−^) emission to the water is calculated as follows (Equation (1)) [27,28]:

PO_4_^3−^ = 0.01 × (P (kg)), (1)

where P is amount of phosphorus applied (kg).

The air emissions of nitrous oxide (N_2_O) and ammonia (NH_3_) associated with fertilization are quantified as follows (Equations (2) and (3)) [27,28]:

N_2_O = 0.0125 × (N (kg)), (2)

NH_3_ = a × (N (kg)). (3)

The NH_3_ emission into the air; therefore, varies according to the nitrogen dose and “a” coefficient. Coefficient of “a” represents the nature of the products applied to the soil and varies based on the type of fertilizer. This coefficient is 8% for urea and ammonium nitrate and 2% for ammonia [28].

Hemp production through photosynthesis causes carbon to be sequestered as long-term storage of atmospheric CO_2_. Since this locked-in CO_2_ has been stored in the hemp stalk for its lifespan, it should be considered as a negative atmospheric CO_2_ emission. Carbon sequestration of hemp stalk was considered to be 1.84 kg of CO_2_/kg [16].

Table 2 shows the physical and hygrothermal properties of studied hemp shiv.

#### 2.2.3. Sol-Gel Coating

**Figure 2:**
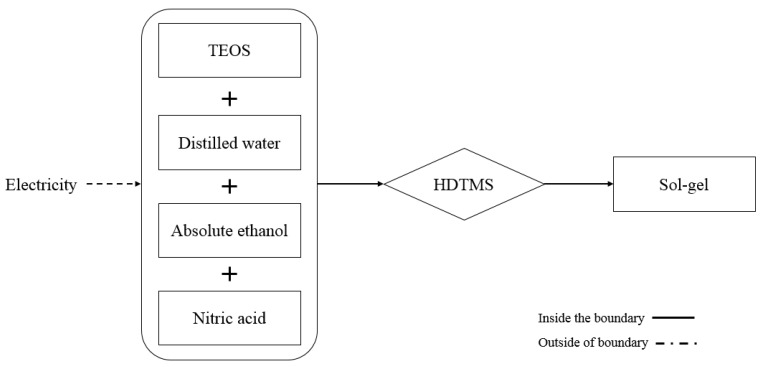
Sol-gel coating production at lab-scale. TEOS: tetraethyl orthosilicate, HDTMS: Hexadecyltrimethoxysilane.

The following steps of the sol-gel coating production at lab-scale were considered during the analysis (Figure 2):

Data for sol-gel coating were compiled from the laboratory of the Department of Architecture and Civil Engineering, University of Bath. In this study, silica sol-gel coating functionalized with a hydrophobic agent was used. Sol-gel coating that was used in this study was synthesised by hydrolysis and condensation of tetraethyl orthosilicate (TEOS, 98%), in ethanol and water. Nitric acid was used to catalyse the reaction. TEOS was added to a mixture of distilled water, nitric acid and ethanol, in order to prepare sol-gel coating material. Hexadecyltrimethoxysilane (HDTMS, 85%), as the hydrophobic agent, was added to the above mixture. The produced sol was stirred for around 2 h and then aged for 48 h in a closed container at room temperature [11,32]. At this stage the sol-gel coating is ready to be applied. After 10 min dipping in coating material, hemp shiv was moved to a petri dish, and left for 1 h at room temperature. The coated hemp shiv was dried at 80 °C for 1 h more.

#### 2.2.4. Bio-Based Composites

Treated hemp shiv as a bio-composite in a hypothetical building wall was compared to two other hypothetical walls, including a wall with untreated hemp shiv and a cavity wall as a reference wall. The dimensions of the walls were calculated in order to have a U-value of 0.15 W/m^2^.K. Table 3 shows the characteristics of the walls of interest in this study, along with their materials used. Dimension and thermal properties of three walls (reference wall, untreated and treated hempcrete walls) are shown in Supplementary Material (Table S1).

Table 3 shows the physical and hygrothermal properties of studied hemp shiv.

The wall with bio-composite was made up of treated and untreated hemp shiv, bio-based binder and water. A timber frame and steel fixings were used as the load-bearing structural elements.

### 2.3. Life Cycle Impact Assessment

Four products are produced after harvesting and processing the hemp plant, which are: hemp seed, hemp fibre, hemp shiv and dust. Economical and mass allocations were exerted to allocate the corresponding environmental impact on each product. The economic and mass allocation coefficients for each product are based on their economic values and weights and are calculated as follows (Equations (4) and (5)):

(4) $${\mathrm{EA}}_{\mathrm{HS}}=\frac{\left(P_{\mathrm{HS}}\times M_{\mathrm{HS}}\right)}{\left(P_{S}\times M_{S})+(P_{F}\times M_{F})+(P_{\mathrm{HS}}\times M_{\mathrm{HS}})+(P_{D}\times M_{D}\right)},$$

(5) $${\mathrm{MA}}_{\mathrm{HS}}=\frac{M_{\mathrm{HS}}}{M_{S}+M_{F}+M_{\mathrm{HS}}+M_{D}},$$

where EA_HS_ and MA_HS_ are economic and mass allocation coefficients of hemp shiv. P_HS_ is the price of 1 kg hemp shiv (€/kg). M_HS_ is mass of hemp shiv produced in one hectare (kg/ha). F, S and D refer to hemp fibre, seed and dust, respectively. Economic and mass allocation for hemp fibre, seed and dust are calculated the same as hemp shiv.

The prices of the hemp coproducts are variable, and they depend on several factors. Considering French market, the average prices were considered to be 0.7 €/kg for the shiv [33], 2.0 €/kg for the hemp fibre, 0.06 €/kg for dust and 2 €/kg for seed [34]. This study presents the results based on two allocation methods (mass and economic) and provides the details of economic and mass allocation for all outputs in the Supplementary Material (Equations (S1)–(S8), and Table S2), this will allow other researchers to recalculate the results based on alternate prices and compare with these results.

In order to analyse the regionalised environmental impacts of hemp shiv and sol-gel coating production, ReCiPe endpoint methodology was used at LCIA step [35]. Damage to terrestrial ecosystem was conducted at damage assessment step, using impacts of land use, terrestrial acidification, photochemical ozone formation, terrestrial ecotoxicity, water use and global warming. Since this regionalised model only considered damage to the terrestrial ecosystem, aggregation impacts over marine, freshwater and terrestrial ecosystems were not considered. Damage to terrestrial ecosystems was expressed by PDF m^2^ y/kg. Characterisation factors for water use, acidification and photochemical ozone formation were applied on a country level. Relevant ecoregions of hemp production were selected in order to calculate the site-specific CF of land use. Since ReCiPe does not cover regionalised CFs for global warming and ecotoxicity; generic CFs were applied for these impact categories. Tables S3–S6 in the Supplementary Material show the regionalised CFs for this study. Environmental impacts of applying treated and untreated hemp shiv in a wall and its comparison with a cavity wall were assessed using four impact assessment methods, including: IPCC 2013 GWP 100a [36], IMPACT 2002+ [37], ReCiPe 2016 Endpoint (H), and CML-IA baseline [38]. Considering choices like time and effects of future technologies, there are three cultural perspectives for ReCiPe method, including individualist (short term), hierarchist (the most common model in scientific projects) and egalitarian (long term) [35]. This study uses Hierarchist perspective of ReCiPe.

### 2.4. End-Of-Life Scenario

Since hemp concrete is relatively novel material, its end-of-life is not well understood. In this study a sensitivity analysis was considered for various end-of-life scenarios, taking into account landfilling and composting. As its recycling has not yet been developed, it was not considered as a scenario. Disposing of the crushed materials in a landfill is the most commonly-used method. For landfilling, a distance of 15 km was considered from the construction place to the landfill.

## 3. Results and Discussion

**Figure 3:**
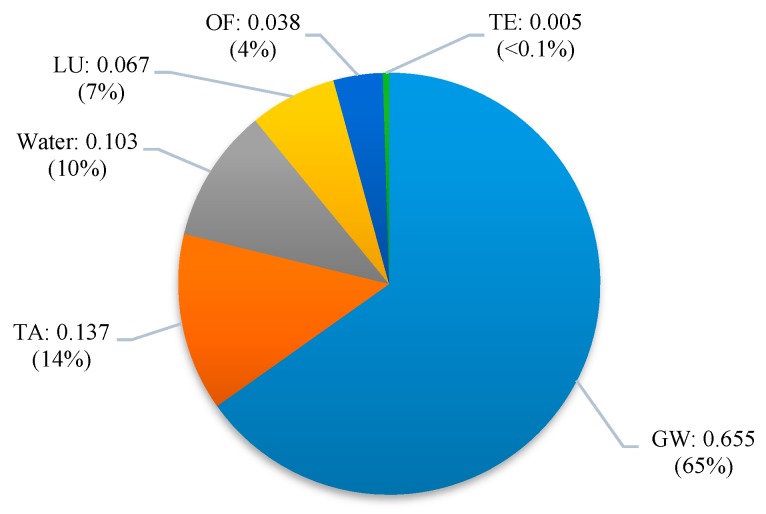
Terrestrial ecosystem damage (PDF m^2^ y/kg) from the production of 1 kg of sol-gel coating, based on the hierarchist perspective. Global warming (GW), ozone formation (OF), terrestrial acidification (TA), terrestrial ecotoxicity (TE), land use (LU).

Figure 3 shows the damage assessment results of 1 kg of sol-gel coating material production, based on regionalised CFs, according to the hierarchist perspective. Terrestrial biodiversity loss of 1 kg of sol-gel coating material production based on all three perspectives is shown in the Supplementary Material, Table S7.

The total damage to the ecosystem from production of 1 kg of sol-gel coating based on the hierarchist perspectives was 1.01 PDF m^2^ y/kg, followed by 0.39 and 5.458 PDF m^2^ y/kg for the individualist, and egalitarian perspectives. Global warming had the highest contribution to the terrestrial biodiversity damage among other environmental impacts. Terrestrial acidification contributed 38% of damage to terrestrial ecosystem based on individualist perspective, followed by global warming 35% (Supplementary Materials, Table S7). Using the hierarchist perspective, global warming was the main contributor (65%), followed by terrestrial acidification 14%. Regarding the egalitarian perspective, global warming was the main contributor at 94%, followed by terrestrial acidification, with only 2%. There are few studies on LCA of sol-gel coatings in the literature, and because of confidentiality issues relating to the chemicals used, their inventory and impact assessment results have not been reported [39]. 

Regarding the contribution of material inputs, TEOS is the environmental hotspot for all impact categories. TEOS as a silica source in the sol-gel material production process is hydrolysed and condensed to make a silicon dioxide network [32]. Looking inside the TEOS production process shows that silicon tetrachloride has the highest impact. Ethanol and electricity were the second and third contributor to total damage to terrestrial ecosystem after TEOS. The contribution of materials in the environmental damage of 1 kg sol-gel coating was shown in the Supplementary Material, Figure S1. Although bio-based coating materials have attracted a lot of attention due to their low environmental impacts, their production cost at the commercialisation level should be considered for future studies.

Table 4 shows the total damage to terrestrial biodiversity of 1 kg hemp shiv production.

Among the environmental impacts, global warming has a negative impact resulting from the sequestered carbon during hemp production (agricultural stage). In spite of the carbon sequestration of hemp production, the agricultural stage is the main contributor to all of the impact categories for hemp shiv production. Looking inside the agricultural stage, it shows that fertiliser application is the hotspot. It is linked to nitrogen consumption and emission of dinitrogen monoxide to the air. Land use impact is the second contributor to total damage to terrestrial ecosystem. Total damage to terrestrial biodiversity of 1 kg hemp shiv production based on all three perspectives is shown in the Supplementary Material, Tables S8–S9.

Hemp plant growth requires a minimum amount of nitrogen, and crop yields and its quality will vary upon nitrogen consumption. Although a higher amount of nitrogen application leads to greater yields, there is no linear trend between nitrogen application and yield, and nitrogen fertilising should be stopped at its economic optimum.

As it can be seen from Table 4, depending on allocation methods, the results vary. The environmental damage associated with mass allocation is greater than those analysed based on economic allocation. Using economic allocation, 32% of all environmental impacts of hemp straw belong to hemp shiv, while for mass allocation its share is 47%. Total yield of 1 ha of hemp production is 8000 kg, including 2745 kg hemp fibre, 3765 kg hemp shiv and 1490 kg dust. As it is recommended by the International Organization for Standardization (ISO 14044) [40], several methods can be applied for allocation (such as mass and economic), but it does not specify a preference for the selection of one particular method. Although the mass allocation method does not require collection and assessment of large amounts of data, it only considers the mass data and there are some debates as to the appropriateness of this method for LCA [41]. This paper reported that the results of implication of mass allocation by over-simplification are purely dependent on the mass relations of the outputs. For example, in a case with a large amount of waste output, the environmental impact of the main products will be low [41,42]. Based on the advantages and disadvantages of mass and economic allocation methods, it is recommended to report both related results and give details of the products (mass contribution and price), so that other researchers are able to update the results and make their own comparisons (see the Supplementary Material, Table S2).

The carbon footprint of functional unit of 1 kg hemp shiv was calculated as −1.55 kg CO_2_-eq/kg and −1.63 kg CO_2_-eq/kg, based on mass and economic allocation, respectively. Carbon footprint of common insulation materials for building has been reported as 7.336 kg CO_2_-eq/kg for expanded polystyrene (EPS) foam slab, 1.511 CO_2_eq/kg for rock wool, 6.788 CO_2_-eq/kg for polyurethane rigid foam, 0.807 kg CO_2_-eq/kg for cork slab, 1.831 kg CO_2_-eq/kg for cellulose fibre and 0.124 kg CO_2_-eq/kg for wood wool [43,44]. Using bio-based materials such as hemp shiv with negative environmental impacts is a promising sustainable building material compared to traditional and synthetic materials.

### 3.1. Regionalised LCA Results

**Figure 4:**
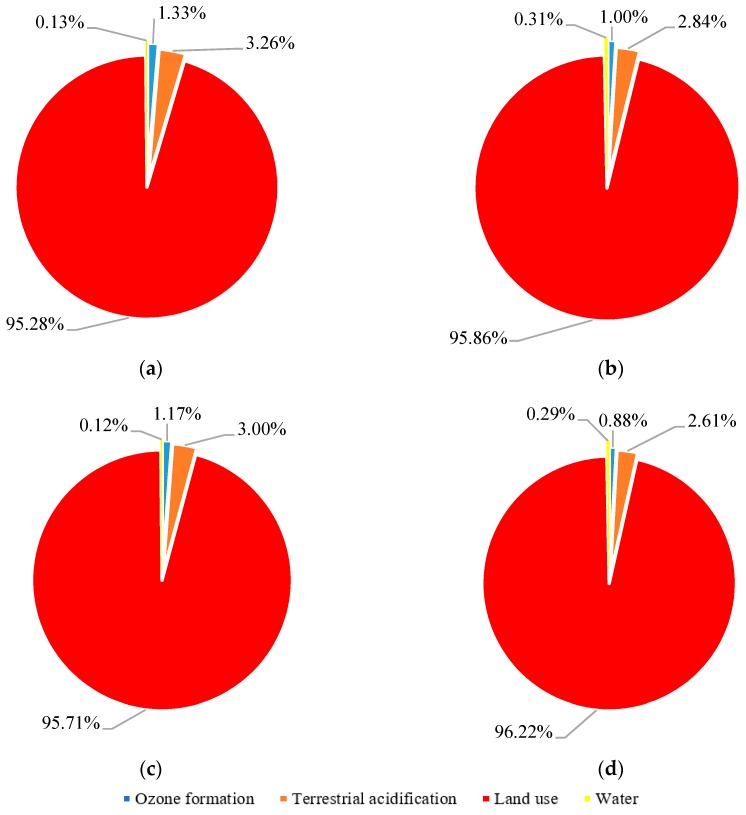
Terrestrial biodiversity of 1 kg of hemp shiv, based on regionalised and generic (world average) CFs for hierarchist perspective. (**a**): Regionalized values and economic allocation; (**b**): Generic values and economic allocation; (**c**): Regionalized values and mass allocation; (**d**): Generic values and mass allocation.

Figure 4 shows the total damage to terrestrial biodiversity by using 1 kg of hemp shiv, based on regionalised and generic (world average) CFs for the hierarchist perspective. Since global warming and terrestrial ecotoxicity results are purely based on generic values, the results of these impact categories were excluded in Figure 4.

The difference between Figure 4a,c, which comes from using different allocation methods, is largely because the environmental damage associated with mass allocation is greater than those analysed based on economic allocation. This difference has been shown between Figure 4b,d for generic results as well. Since regionalised characterisation factors for three perspectives are same, the comparison of the results is shown based on one perspective (hierarchist). Regardless of generic impact categories, land use is the main driver for both allocation methods (mass and economic). The damage to terrestrial biodiversity based on regionalised characterisation factors shows greater values than generic ones. The main differences come from the impact of land use. Looking inside the site-specific characterisation factors of land use [45] shows that the median regionalised characterisation factor for land occupation of annual crop (e.g., hemp) is 0.76 biodiversity damage potential (BDP), while the generic value (world average) is 0.60 BDP. Regionalised characterisation factors of land use have been selected based on the world wildlife (WWF) ecoregion map [46]. Based on the geographical location of the studied farms, WWF biome 4 (temperate broadleaf forest) was chosen and its corresponding characterisation factors were applied. Land use change is a combination of three land use impacts: land transformation, land occupation and permanent impact of land use. Since there was no reliable data for site-specific characterisation factors of land transformation and permanent impacts, these two impacts were calculated based on generic values. Terrestrial acidification was the second major contributor among other impact categories (2.61–3.26%). The contribution and difference of the other two impact categories (i.e., ozone formation and water use) were slightly visible because of the large contribution of land use. Comparison of the results of terrestrial acidification based on site-specific and generic data shows that using regionalised characterisation factors increase the terrestrial biodiversity damage by 60%. This difference is 62% for land use. As the results of the comparison show, the use of generic values has a relatively high difference compared with regionalised ones; however, the site-specific characterisation factors were applied from different literature. It is suggested to include site-specific characterisation factors in LCIA methods and in the case of access to regionalized characterization factors, these values are used for analysis.

### 3.2. Comparison of Wall Scenarios

**Figure 5:**
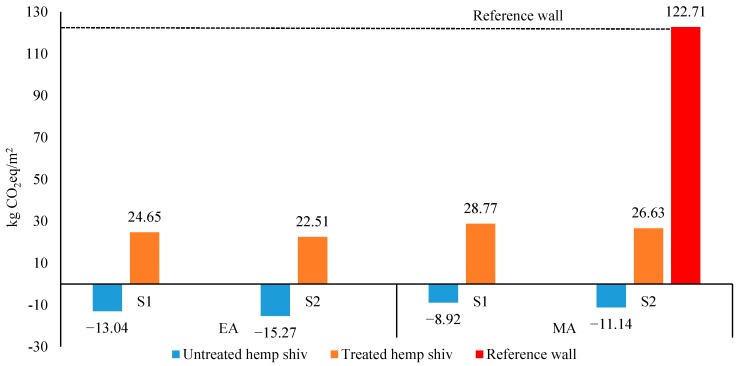
Carbon footprint of 1 m^2^ walls for their entire service life. EA and MA show the results based on economic and mass allocation. S1 and S2 are waste scenarios (S1—composting hemp shiv and landfilling the rest, S2—landfilling all the materials).

Carbon footprint of one square meter of a wall, using treated and untreated hemp shiv was calculated using the IPCC 2013 GWP 100a method [36]. Using economic allocation, the total carbon footprint of one m^2^ of treated hempcrete wall (for its entire service life) is 24.65 kg CO_2_-eq and 22.51 kg CO_2_-eq based on end-of-life treatment as composting and landfilling, respectively. The results based on mass allocation and untreated hempcrete wall are shown in Figure 5.

The sensitivity analysis, considering different allocation methods and waste scenarios, show variation among results (Figure 5). As it is presented in Table 3, the main materials constructing the reference wall include clay brick, insulation panel, concrete block, gypsum plasterboard and stucco. Hempcrete walls use treated/untreated hemp shiv, bio-based binder (Tradical ThermO, Besançon, Bourgogne-Franche-Comté, France), timber frame and steel fastening. Hemp shiv is the main driver of greenhouse gas emissions among the materials of two studied walls (hempcrete walls). Since in the aggregate of untreated hempcrete wall, there is no coating material, it only uses hemp shiv and the negative environmental impact of hemp shiv reduces the carbon footprint of a untreated wall to a negative value. Use of treated hemp shiv decreases the water consumption of hempcrete mix (i.e., treated hempcrete used less 20 L water per 20 kg of hemp shiv than untreated hempcrete), although this difference does not affect the total carbon footprint of this wall compared to the untreated wall. Binder consumption of both walls (treated and untreated) is 91.2 kg/m^2^ wall and its carbon footprint balance is same (53.34 kg CO_2_eq/m^2^ wall).

Table 5 shows the carbon footprint of the treated and untreated hempcrete walls of the current study compared to similar studies.

The current study considered a U-value of 0.15 W/m^2^∙K for all scenarios, which requires a thicker wall compared with similar studies. The calculation of wall thickness for wall scenarios is shown in Supplementary Material (Table S1).

**Figure 6:**
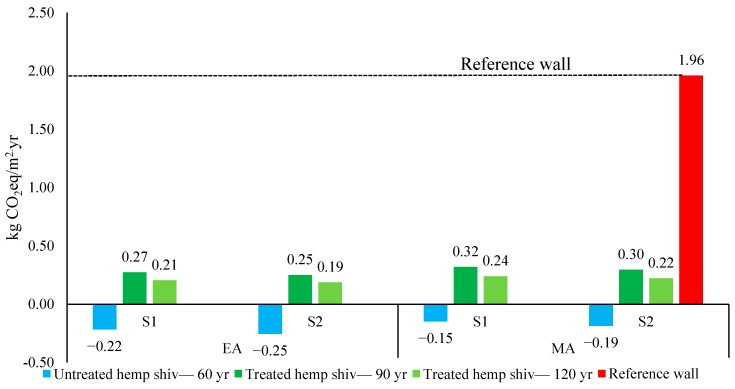
Carbon footprint of 1 m^2^ hempcrete wall compared to reference wall. EA and MA show the results based on economic and mass allocation. S1 and S2 are waste scenarios (S1—composting hemp shiv and landfilling the rest, S2—landfilling all the materials).

In order to compare the carbon footprint balance of a bio-based insulated wall with a reference wall (with service life of 60 years), a comparison was conducted with a traditional insulated masonry cavity wall with a U-value of 0.15 W/m^2^∙K. Since the service life of hempcrete walls are not similar, the results are based on one-year service life of the wall. A sensitivity analysis was applied to the service life of the walls (Figure 6).

Use of annual-growing bio-based materials, such as hemp shiv, creates a good opportunity to significantly decrease the greenhouse gas emissions of building materials [50]. The carbon that is banked through plant photosynthesis and carbon sequestration is one of the main aspects of the environmental profile of bio-based materials. Complexity of bio-based materials has been reported as one the reason that there is no standardised approach for assessment of biogenic carbon storage [51]. Since carbon sequestration has the highest share in carbon footprint of bio-based materials, use of unstandardized approaches can lead to inaccurate accounting of the carbon balance of these materials.

**Figure 7:**
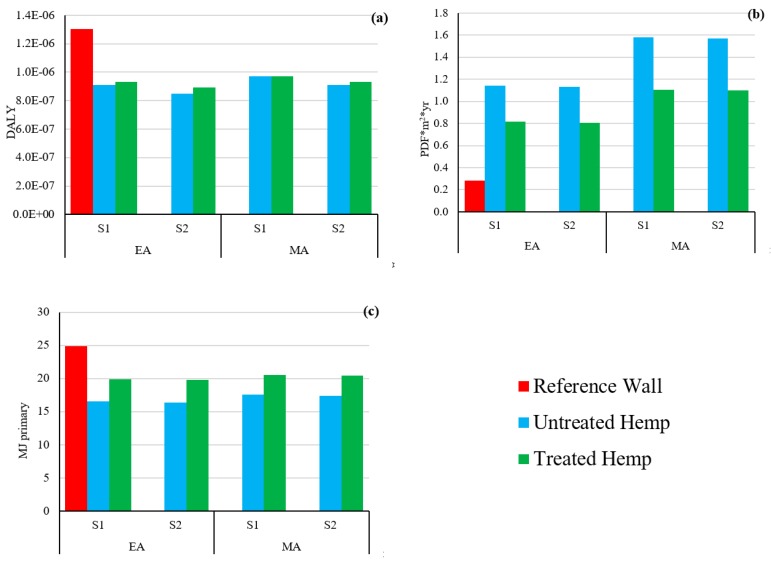
Endpoint environmental impacts of 1 m^2^ hempcrete wall compared to cavity wall. (**a**) Human health, (**b**) ecosystem quality, (**c**) resources. EA and MA show the results based on economic and mass allocation, respectively. S1 and S2 are waste scenarios (S1—composting hemp shiv and landfilling the rest, S2—landfilling all the materials).

Hemp, as one of the most important sources of bio-based insulation fibre and bio-aggregate, when incorporated into a building dramatically reduces its carbon footprint; however, the carbon footprint of treated hempcrete is higher than untreated one because of using coating material. The long-term benefits of using treated hempcrete such as biodegradation delay would make it a promising option as a sustainable bio-based building material, which can be focused in future studies. In order to compare the results of environmental impacts of different wall scenarios based on other impact assessment methods, a sensitivity analysis was applied. Figure 7 shows the results of 1 m^2^ of wall using the IMPACT 2002+ method.

Considering damage to human health (Figure 7a), both hempcrete walls (treated and untreated) have a lower impact than the reference wall. Looking inside the reference wall, it shows that production of insulation panel and solid brick are main contributors to human health damage. The reference wall had lower damage to the ecosystem (Figure 7b) compared with the hempcrete walls. The higher impacts of hempcrete walls (based on ecosystem quality) come from land occupation of the hemp production process (67–76%). Comparison of walls based on damage to resources (Figure 7c), indicates that reference wall has the highest environmental impact and untreated wall has the lowest one. The results of a comparison of the walls based on ReCiPe 2016 midpoint and endpoint (H) and CML-IA baseline methods are reported in Supplementary Material (Tables S10–S12). Based on the results of this study, hempcrete can be considered as a sustainable building material compared to traditional and synthetic materials. Renewability and availability of the sources of these materials would make lighter environmental impact among its life cycle and have less pressure on natural resources. 

Literature shows that bio-based material (e.g., bio-based composites) lead to positive consequences in specific areas, relating to greenhouse gas emissions, terrestrial acidification, fossil fuel demand and water eutrophication [52]. Using LCA on bio-based materials helps to identify the consequences of material selection in the environmental profile of a product/service, moreover it guides eco-designers to choose the type of material that is desirable for improving the environmental burdens of the final product. For instance, hempcrete provides lightweight components (62.32% lighter than cavity wall), which cause lowering of the carbon footprint and even lower energy demands on the construction envelope. However, for the construction sector, it is essential that the cost-effectiveness, improvement of thermal conductivity and mechanical properties, be considered when introducing novel bio-based materials.

Sensitivity analysis on allocation method and service life of hempcrete walls was applied, although when it comes to end-of-life, it introduces uncertainty through different scenarios [53] because the end-of-life scenario for hempcrete is not yet established. Therefore, uncertainties from modelling of the processes and particularly at the end-of-life stage should be considered in further research. One of the limitations of this study is a lack of uncertainty analysis for regionalised characterisation factors. Since there was no value choice for all regionalised impact categories in one study and several studies were used to calculate them, uncertainty of characterisation factors was excluded. In order to provide the results with a reliable level of confidence, more research is needed to conduct different sources of uncertainty, and considering different climate conditions, when applying a regionalised LCA model.

## 4. Conclusions

The goal of the presented study was to identify and quantify the environmental impacts of treating hemp shiv, with sol-gel based coating, as a bio-based material in a building wall. Hempcrete as a bio-based insulation material compares favourably with the environmental impact of synthetic/mineral construction materials throughout its carbon sequestration effect. In this study, regionalised terrestrial ecosystem damage of hemp shiv production was analysed through a regionalised life cycle assessment model. Sensitivity analysis of allocation methods was conducted on the results of hemp shiv production. The results in the case of hemp shiv show important differences between regionalised and generic values. Regionalised LCA modelling makes it possible for decision-makers at the country-scale to access more consistent site-specific information with more relevance of resulting calculations, so they can make more realistic and accurate decisions rather than relying on generic assessments. However, it is recommended that more studies be applied to assess the regionalised characterisation factors of land transformation, permanent impacts and different substances of terrestrial ecotoxicity in order to have a comprehensive regionalised analysis of terrestrial ecosystem damage.

Carbon footprint balance of bio-based insulated walls using treated and untreated hemp shiv, using sol gel based coating, was compared to a reference cavity wall, using a U-value of 0.15 W/m^2^∙K for all scenarios. Results have indicated that applying bio-based insulation materials as untreated hempcrete cause a negative carbon balance in the building envelope. However, using treated hempcrete will increase the lifespan of the wall. Low environmental impacts, renewability and availability of the sources of hempcrete as a bio-based material make it a sustainable building material compared to traditional and synthetic materials.

## Figures and Tables

**Table 1:** Hemp plant and hemp shiv inventory data.

Material	Unit	Amount
**Input**	-	-
Seed	kg/ha	55.0
Fertiliser	-	-
Nitrogen (N)	kg/ha	85.0
Phosphate (P_2_O_5_)	kg/ha	65.0
Potassium (K_2_O)	kg/ha	125.0
Agricultural machinery	kg/ha	17.0
Diesel	kg/ha	74.0
Electricity	kWh/ha	360.0
Transport	t.km/ha	90.0
**Output**	-	-
Straw	kg/ha	8000.0
Shiv	kg/ha	3764.7
Fibre	kg/ha	2745.1
Hemp dust	kg/ha	1490.2
Seed	kg/ha	1020.0

**Table 2:** Physical and hygrothermal properties of studied materials.

Characteristic	Unit	Value	Reference
Bulk density	kg/m^3^	90–110	[29]
Absolute density	kg/m^3^	1450	[29]
Porosity	%	76.67	[29]
Moisture buffer value	g/(m^2^%RH) *	2.07	[30]
Thermal conductivity	W/m.K	0.05–0.06	[31]

* RH: Relative humidity.

**Table 3:** Characteristics of one m^2^ of walls and materials used.

Scenario	Material	Unit	Value	Comment
Reference wall	Solid brick	kg	183.6	Clay brick| Market for| Cut-off, U
	Air cavity	kg	-	None
	Eco-therm insulation	kg	11.6	Kingspan Insulation panel
	Cement block	kg	200.0	Concrete block| Market for| Cut-off, U
	Gypsum plasterboard	kg	8.7	Gypsum plasterboard| Market for| Cut-off, U
	Gypsum plaster	kg	2.1	Stucco| Market for| Cut-off, U
Treated bio-composite wall	Treated hemp shiv	kg	61.8	82% Hemp shiv and 18% sol-gel-based coating
	Water	kg	101.3	Tap water| Market for | Cut-off, U
	Bio-based binder	kg	91.2	Tradical ThermO (Besançon, Bourgogne-Franche-Comté, France), modelled on SimaPro
	Timber frame	kg	9.3	Plywood, for outdoor use| Market for| Cut-off, U
	Steel fastening	kg	5.0	Steel, unalloyed| Market for| Cut-off, U
Untreated bio-composite wall	Untreated hemp shiv	kg	50.7	100% Hemp shiv
	Water	kg	152.0	Tap water| Market for | Cut-off, U
	Bio-based binder	kg	91.2	Tradical ThermO (Besançon, Bourgogne-Franche-Comté, France), modelled on SimaPro
	Timber frame	kg	9.3	Plywood, for outdoor use| Market for| Cut-off, U
	Steel fastening	kg	5.0	Steel, unalloyed| Market for| Cut-off, U

**Table 4:** Terrestrial ecosystem damage (PDF m^2^ y/kg) from the production of 1 kg of hemp shiv, based on hierarchist perspective. EA and MA show the results based on economic and mass allocation, respectively.

Impact Category (PDF m^2^ y/kg)	Value	
	EA	MA
Land use	0.532	0.863
Terrestrial acidification	0.018	0.027
Ozone formation	0.007	0.011
Terrestrial ecotoxicity	0.001	0.001
Water	0.001	0.001
Global warming	−0.308	−0.292

**Table 5:** Comparison of the carbon footprint of similar studies on hempcrete walls with the current study.

Study	Wall Thickness (mm)	U-Value (W/m^2^∙K)	Carbon Footprint (kg CO_2_eq)	Reference
Boutin et al.	260	0.42	−35.53	[33]
Ip and Miller	300	0.19	−36.08	[47]
Pretot et al.	240 + 10^*^	0.36	−1.60	[48]
Arrigoni et al.	250	0.27	−12.09	[49]
Untreated hempcrete	507	0.15	−15.27 to −8.92	Current study
Treated hempcrete	507	0.15	22.51 to 28.77	Current study

* This study has 10 mm of coating.

## Supplementary Materials

The following are available online at https://www.mdpi.com/1996-1944/12/18/2987/s1, Equations (S1)–(S8), Figure S1: Terrestrial ecosystem damage (PDF m^2^ y/kg) from the production of 1 kg of sol-gel, based on the hierarchist perspective, Table S1: Dimension and thermal properties of 1 m^2^ of three walls (reference wall, untreated and treated hempcrete walls), Table S2: Terrestrial ecosystem damage (PDF m^2^ y/kg) from the production of 1 kg of sol-gel, based on three perspectives, Table S3: Endpoint characterization factors (PDF m^2^ y/kg) of France and England for photochemical ozone formation, Table S4: Region-specific endpoint characterization factors (PDF m^2^ y/kg) for terrestrial acidification, Table S5: Endpoint characterization factors (PDF m^2^ y/m^2^) for land use (land occupation) of France and England, Table S6: Endpoint characterization factors (PDF m^2^ y/m^2^) for water use of France and England, Table S7: Terrestrial ecosystem damage (PDF m^2^ y/kg) from the production of 1 kg of sol-gel, based on three perspectives; Table S8: Terrestrial ecosystem damage (PDF m^2^ y/kg) from the production of 1 kg of hemp shiv, based on three perspectives, Table S9: Comparison of regionalized and generic results of terrestrial ecosystem damage (PDF m^2^ y/kg) from the production of 1 kg of hemp shiv, based on three perspectives, Table S10: Life cycle impact assessment of 1 m^2^ wall, using ReCiPe Mimpoint (H) method, Table S11: Damage assessment of 1 m^2^ wall, using ReCiPe Endpoint (H) method, Table S12: Life cycle impact assessment of one m^2^ wall, using CML-IA baseline method.

## Abbreviations

BDP	Biodiversity Damage Potential
CF	Characterisation Factor
DALY	Disability-Adjusted Life Year
EA	Economic Allocation
EPS	Expanded Polystyrene
FU	Functional Unit
GW	Global Warming
GWP	Global Warming Potential
HDTMS	Hexadecyltrimethoxysilane
Hempcrete	Hemp-Concrete
ISO	International Organization for Standardization
LCA	Life Cycle Assessment
LCI	Life Cycle Inventory
LCIA	Life Cycle Impact Assessment
LU	Land Use
IPCC	Intergovernmental Panel on Climate Change
K_2_O	Potassium
MA	Mass Allocation
N	Nitrogen
N_2_O	Nitrous Oxide
NH_3_	Ammonia
NO_3_^−^	Nitrate
OF	Ozone Formation
PDF	Potentially Disappeared Fraction of Species
PO_4_^3−^	Phosphate
P_2_O_5_	Phosphate
PVC	Polyvinyl Chloride
RH	Relative Humidity
TA	Terrestrial Acidification
TE	Terrestrial Ecotoxicity
TEOS	Tetraethyl Orthosilicate
U-value	Thermal Transmittance
WWF	World Wildlife
