# Supplementary material for: Regionalised Life Cycle Assessment of Bio-Based Materials in Construction; the Case of Hemp Shiv Treated with Sol-Gel Coatings
Source: Materials (Basel). 2019 Sep 15;12(18):2987. doi: 10.3390/ma12182987 (PMC6766272; doi:10.3390/ma12182987)
Supplement: Supplementary file 1 [file materials-12-02987-s001.pdf]

materials

Article

Regionalised Life Cycle Assessment of Bio-Based Materials in Construction; the Case of Hemp Shiv Treated with Sol-Gel Coatings

Mohammad Davoud Heidari [1,2], Michael Lawrence [3], Pierre Blanchet [2] and Ben Amor [1,*]

Table S1. Dimension and thermal properties of 1 m² of three walls (reference wall, untreated and treated hempcrete walls).

Material	Thickness (mm)	λ (W/m.K)	R (m².K/W)	U (W/m².K)
Reference wall	390	0.06	6.67	0.15
Solid brick	102	0.88	0.12	8.64
Air cavity inc metal wall ties	50	0.27	0.19	5.40
Eco therm cavity insulation	122	0.02	5.55	0.18
Cement block	100	0.13	0.74	1.34
Gypsum plasterboard	13	0.20	0.06	15.53
Gypsum plaster	3	0.43	0.01	143.67
Untreated hempcrete wall	507	0.076	6.67	0.15
Treated hempcrete wall	507	0.076	6.67	0.15

Allocation Method

When there is more than one output or there are some co-products, the ISO 14044 standard recommends dividing the inputs and outputs of that process among different products. This term is called allocation. As it is recommended by ISO 14044 [1], several methods can be applied for allocation (mass and economic), but it does not identify the preference of one allocation method. Mass allocation considers physical relationships between the different products based on their weights, while economic allocation is according to respective economic values of different products.

Four products are produced after harvesting and processing harvested hemp plant, including: hemp seed, hemp fibre, hemp shiv and dust. The mass allocation coefficients for each product are in accordance to their weights and are calculated as follows (Equations (S1)–(S4)):

$$\mathrm{MA_S} = \frac{\mathrm{M_S}}{\mathrm{M_S + M_F + M_{HS} + M_D}}, \tag{S1}$$

$$\mathrm{MA_F} = \frac{\mathrm{M_F}}{\mathrm{M_S + M_F + M_{HS} + M_D}}, \tag{S2}$$

$$\mathrm{MA_{HS}} = \frac{\mathrm{M_{HS}}}{\mathrm{M_S + M_F + M_{HS} + M_D}}, \tag{S3}$$

$$\mathrm{MA_D} = \frac{\mathrm{M_D}}{\mathrm{M_S + M_F + M_{HS} + M_D}}, \tag{S4}$$

where MA is the mass allocation coefficient of each product (S, F, HS and D refer to hemp seed, fibre, shiv and dust, respectively). M_S is the mass of hemp seed produced in 1 ha (kg/ha). M_F, M_{HS} and M_D are the mass of hemp fibre, shiv and dust, respectively.

The economic allocation coefficients for each product are in accordance to their economic values and are calculated as follows (Equations. (S5)–(S8)):

$$\mathrm{EA_S} = \frac{(\mathrm{P_S \times M_S})}{(\mathrm{P_S \times M_S}) + (\mathrm{P_F \times M_F}) + (\mathrm{P_{HS} \times M_{HS}}) + (\mathrm{P_D \times M_D})}, \tag{S5}$$

$$EA_F = \frac{(P_F \times M_F)}{(P_S \times M_S) + (P_F \times M_F) + (P_{HS} \times M_{HS}) + (P_D \times M_D)}, \quad (S6)$$

$$EA_{HS} = \frac{(P_{HS} \times M_{HS})}{(P_S \times M_S) + (P_F \times M_F) + (P_{HS} \times M_{HS}) + (P_D \times M_D)}, \quad (S7)$$

$$EA_D = \frac{(P_D \times M_D)}{(P_S \times M_S) + (P_F \times M_F) + (P_{HS} \times M_{HS}) + (P_D \times M_D)}, \quad (S8)$$

where EA is economic allocation coefficient of each product (S, F, HS and D refer to hemp seed, fibre, shiv and dust respectively). P_S is the price of 1 kg hemp seed (€/kg). P_F, P_{HS} and P_D are price (€/kg) of fibre, shiv and dust, respectively.

The prices of the hemp coproducts are variable, and they depend on several factors. Considering the French market, the average prices were considered to be 2 €/kg for seed, 2.0 €/kg for the hemp fibre [2], 0.7 €/kg for the shiv [3] and 0.06 €/kg for dust [2]. Table S1 Shows the details of economic and mass allocation for all outputs in order to be able to recalculate the results based on new prices.

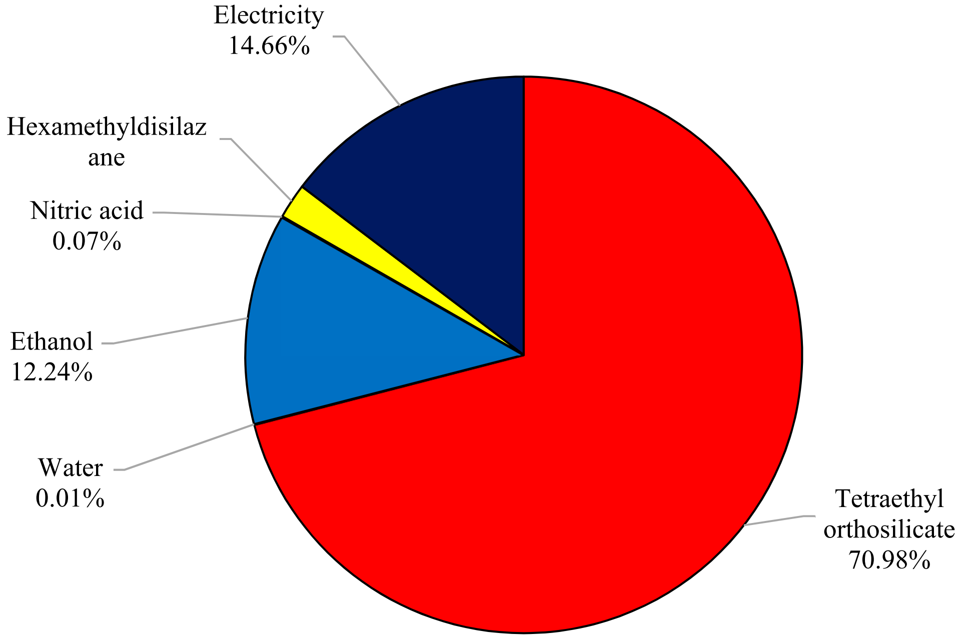

Figure S1. Terrestrial ecosystem damage (PDF m^2 y/kg) from the production of 1 kg of sol-gel, based on the hierarchist perspective.

Table S2. Terrestrial ecosystem damage (PDF m^2 y/kg) from the production of 1 kg of sol-gel, based on three perspectives.

Component	Abbreviation	Unit	Value	Reference
Mass allocation	-	-	-	-
Mass of hemp seed	M_S	kg/ha	1020.00	Current study
Mass of hemp fibre	M_F	kg/ha	2745.10	Current study
Mass of hemp shiv	M_{HS}	kg/ha	3764.71	Current study
Mass of dust	M_D	kg/ha	1490.20	Current study
Mass allocation coefficient of hemp seed	MA_S	-	11.31	Calculated
Mass allocation coefficient of hemp fibre	MA_F	-	30.43	Calculated
Mass allocation coefficient of hemp shiv	MA_{HS}	-	41.74	Calculated
Mass allocation coefficient of dust	MA_D	-	16.52	Calculated
Economic allocation	-	-	-	-
Price of hemp seed	P_S	€/kg	2.00	[2]
Price of hemp fibre	P_F	€/kg	2.00	[2]
Price of hemp shiv	P_{HS}	€/kg	0.70	[3]
Price of dust	P_D	€/kg	0.06	[2]

Economic allocation coefficient of hemp seed	EA_S	-	19.89	Calculated
Economic allocation coefficient of hemp fibre	EA_F	-	53.54	Calculated
Economic allocation coefficient of hemp shiv	EA_{HS}	-	25.70	Calculated
Economic allocation coefficient of dust	EA_D	-	0.87	Calculated

Life Cycle Impact Assessment

Table S3. Endpoint characterization factors (PDF m^2 y/ kg) of France and England for photochemical ozone formation [4].

Substance	France	England
NMVOC	7.55	7.65
NOx	14.06	3.26

Table S4. Region-specific endpoint characterization factors (PDF m^2 y/kg) for terrestrial acidification [5].

Substance	France	England
NOx	7.98	5.10
NH_3	40.20	16.40
SO_2	20.50	11.90

Table S5. Endpoint characterization factors (PDF m^2 y/m^2) for land use (land occupation) of France and England [6].

Area of protection	land use type	France	England
Terrestrial ecosystems	Forest, not used	0.00	0.00
	Secondary vegetation	0.08	0.08
	Forest, used	0.22	0.22
	Pasture/meadow	0.52	0.52
	Annual crops	0.76	0.76
	Permanent crops	0.02	0.02
	Agroforestry	0.00	0.00
	Artificial areas	0.40	0.40

Table S6. Endpoint characterization factors (PDF m^2 y/m^2) for water use of France and England [7].

Area of protection	Country	Individualist	Hierarchist	Egalitarian
Terrestrial ecosystems	France	0.00	0.16	0.16
	England	0.00	0.12	0.12

Table S7. Terrestrial ecosystem damage (PDF m^2 y/kg) from the production of 1 kg of sol-gel, based on three perspectives.

Impact category	Individualist	Hierarchist	Egalitarian
Global warming	0.148	0.655	5.127
Ozone formation	0.038	0.038	0.038
Terrestrial acidification	0.137	0.137	0.137
Terrestrial ecotoxicity	0.002	0.005	0.005
Land use	0.067	0.067	0.067
Water	0.000	0.103	0.103

Table S8. Terrestrial ecosystem damage (PDF m^2 y/kg) from the production of 1 kg of hemp shiv, based on three perspectives. EA and MA show the results based on economic and mass allocation, respectively.

Impact category	Individualist		Hierarchist		Egalitarian	
	EA	MA	EA	MA	EA	MA
Global warming	–0.058	–0.055	–0.308	–0.292	–2.833	–2.738
Ozone formation	0.007	0.011	0.007	0.011	0.007	0.011

Terrestrial acidification	0.018	0.027	0.018	0.027	0.018	0.027
Terrestrial ecotoxicity	0.001	0.001	0.001	0.001	0.001	0.001
Land use	0.532	0.863	0.532	0.863	0.532	0.863
Water	0.000	0.000	0.001	0.001	0.001	0.001

Table S9. Comparison of regionalized and generic results of terrestrial ecosystem damage (PDF m^2 y/kg) from the production of 1 kg of hemp shiv, based on three perspectives. EA and MA show the results based on economic and mass allocation, respectively.

**Impact category**	**Individualist**				**Hierarchist**				**Egalitarian**			
	EA*		MA**		EA		MA		EA		MA	
Spatial resolution	Regionalized	Generic	Regionalized	Generic	Regionalized	Generic	Regionalized	Generic	Regionalized	Generic	Regionalized	Generic
Global warming	−0.058	−0.058	−0.055	−0.055	−0.308	−0.308	−0.292	−0.292	−2.833	-2.833	−2.738	−2.738
Ozone formation	0.007	0.004	0.011	0.006	0.007	0.004	0.011	0.006	0.007	0.004	0.011	0.006
Terrestrial acidification	0.018	0.013	0.027	0.019	0.018	0.013	0.027	0.019	0.018	0.013	0.027	0.019
Terrestrial ecotoxicity	0.001	0.001	0.001	0.001	0.001	0.001	0.001	0.001	0.001	0.001	0.001	0.001
Land use	0.532	0.424	0.863	0.687	0.532	0.424	0.863	0.687	0.532	0.424	0.863	0.687
Water	0.000	0.000	0.000	0.000	0.001	0.001	0.001	0.002	0.001	0.001	0.001	0.002

Table S10. Life cycle impact assessment of 1 m^2 wall, using ReCiPe midpoint (H) method. EA and MA show the results based on economic and mass allocation. S1 and S2 are waste scenarios (S1—composting hemp shiv and landfilling the rest, S2—landfilling all the materials).

**Damage category**	**Unit**	**Reference Wall**	**Untreated Hemp**				**Treated Hemp**			
			**EA**		**MA**		**EA**		**MA**	
			S1	S2	S1	S2	S1	S2	S1	S2
Global warming	kg CO_2 eq	2.0E+00	−2.0E−01	−2.4E−01	−1.2E−01	−1.7E−01	3.0E−01	2.7E−01	3.5E−01	3.2E−01
Stratospheric ozone depletion	kg CFC11 eq	5.1E−07	2.5E−06	2.2E−06	3.6E−06	3.4E−06	1.8E−06	1.7E−06	2.6E−06	2.4E−06
Ionizing radiation	kBq Co-60 eq	4.7E−02	6.9E−02	6.8E−02	9.2E−02	9.1E−02	7.3E−02	7.3E−02	8.9E−02	8.8E−02
Ozone formation, Human health	kg NOx eq	4.3E−03	2.8E−03	2.8E−03	3.0E−03	2.9E−03	2.7E−03	2.7E−03	2.8E−03	2.8E−03
Fine particulate matter formation	kg PM2.5 eq	2.3E−03	1.7E−03	1.5E−03	1.8E−03	1.6E−03	1.7E−03	1.6E−03	1.8E−03	1.7E−03
Ozone formation, Terrestrial ecosystems	kg NOx eq	4.6E−03	2.9E−03	2.9E−03	3.1E−03	3.0E−03	2.8E−03	2.8E−03	2.9E−03	2.9E−03
Terrestrial acidification	kg SO_2 eq	5.2E−03	4.8E−03	3.6E−03	5.2E−03	4.0E−03	4.6E−03	3.8E−03	4.9E−03	4.1E−03
Freshwater eutrophication	kg P eq	4.1E−04	2.1E−04	2.0E−04	2.3E−04	2.2E−04	3.2E−04	3.2E−04	3.4E−04	3.4E−04
Marine eutrophication	kg N eq	2.8E−05	1.8E−04	1.7E−04	2.8E−04	2.8E−04	1.3E−04	1.3E−04	2.0E−04	2.0E−04
Terrestrial ecotoxicity	kg 1,4-DCB	3.4E+00	6.5E+00	6.5E+00	6.8E+00	6.8E+00	5.1E+00	5.1E+00	5.3E+00	5.3E+00
Freshwater ecotoxicity	kg 1,4-DCB	6.0E−02	1.6E−02	1.6E−02	1.9E−02	1.8E−02	2.1E−02	2.1E−02	2.3E−02	2.3E−02
Marine ecotoxicity	kg 1,4-DCB	7.9E−02	2.6E−02	2.5E−02	2.9E−02	2.8E−02	3.2E−02	3.1E−02	3.4E−02	3.3E−02
Human carcinogenic toxicity	kg 1,4-DCB	1.1E−01	4.5E−02	4.3E−02	4.7E−02	4.5E−02	4.4E−02	4.2E−02	4.5E−02	4.3E−02
Human non-carcinogenic toxicity	kg 1,4-DCB	8.0E−01	4.8E−01	4.7E−01	5.4E−01	5.3E−01	5.9E−01	5.8E−01	6.3E−01	6.2E−01
Land use	m^2a crop eq	6.7E−02	8.7E−01	8.7E−01	1.2E+00	1.2E+00	5.9E−01	5.9E−01	8.4E−01	8.4E−01
Mineral resource scarcity	kg Cu eq	6.3E−02	9.9E−03	9.8E−03	1.0E−02	1.0E−02	7.4E−03	7.3E−03	7.6E−03	7.6E−03

Fossil resource scarcity	kg oil eq	5.1E−01	3.3E−01	3.3E−01	3.5E−01	3.4E−01	4.0E−01	4.0E−01	4.1E−01	4.1E−01
Water consumption	m^3	1.6E−02	8.1E−03	8.3E−03	8.8E−03	8.9E−03	3.2E−02	3.2E−02	3.3E−02	3.3E−02

Table S11. Damage assessment of 1 m^2 wall, using ReCiPe endpoint (H) method. EA and MA show the results based on economic and mass allocation, respectively. S1 and S2 are waste scenarios (S1—composting hemp shiv and landfilling the rest, S2—landfilling all the materials).

**Damage category**	**Unit**	**Reference Wall**	**Untreated Hemp**				**Treated Hemp**			
			**EA**		**MA**		**EA**		MA	
			S1	S2	S1	S2	S1	S2	S1	S2
Human health	DALY	3.9E−06	1.2E−06	1.0E−06	1.3E−06	1.2E−06	1.7E−06	1.6E−06	1.8E−06	1.7E−06
Ecosystems	species.yr	8.5E−09	8.9E−09	8.5E−09	1.3E−08	1.2E−08	8.2E−09	7.9E−09	1.1E−08	1.0E−08
Resources	USD2013	1.8E−01	1.2E−01	1.2E−01	1.3E−01	1.3E−01	1.4E−01	1.4E−01	1.5E−01	1.5E−01

Table S12. Life cycle impact assessment of 1 m^2 wall, using CML-IA baseline method. EA and MA show the results based on economic and mass allocation, respectively. S1 and S2 are waste scenarios (S1—composting hemp shiv and landfilling the rest, S2—landfilling all the materials).

**Damage category**	**Unit**	**Reference Wall**	**Untreated Hemp**				**Treated Hemp**			
			**EA**		**MA**		**EA**		MA	
			S1	S2	S1	S2	S1	S2	S1	S2
Abiotic depletion	kg Sb eq	5.9E−06	2.0E−06	1.9E−06	2.4E−06	2.4E−06	2.6E−06	2.6E−06	2.9E−06	2.9E−06
Abiotic depletion (fossil fuels)	MJ	2.2E+01	1.4E+01	1.4E+01	1.5E+01	1.5E+01	1.7E+01	1.7E+01	1.8E+01	1.7E+01
Global warming (GWP100a)	kg CO2 eq	2.0E+00	−2.2E−01	−2.6E−01	−1.5E−01	−1.9E−01	2.7E−01	2.5E−01	3.2E−01	2.9E−01
Ozone layer depletion (ODP)	kg CFC-11 eq	9.7E−08	1.5E−07	1.5E−07	1.6E−07	1.6E−07	1.6E−07	1.6E−07	1.6E−07	1.6E−07
Human toxicity	kg 1,4-DB eq	7.4E−01	3.8E−01	3.7E−01	4.1E−01	4.0E−01	4.1E−01	4.1E−01	4.3E−01	4.3E−01
Fresh water aquatic ecotox.	kg 1,4-DB eq	6.3E−01	1.9E−01	1.8E−01	2.1E−01	2.0E−01	2.5E−01	2.5E−01	2.7E−01	2.6E−01
Marine aquatic ecotoxicity	kg 1,4-DB eq	2.9E+03	5.7E+02	5.5E+02	6.2E+02	6.0E+02	8.7E+02	8.5E+02	9.0E+02	8.9E+02
Terrestrial ecotoxicity	kg 1,4-DB eq	2.8E−03	3.6E−03	3.5E−03	4.7E−03	4.7E−03	3.3E−03	3.2E−03	4.0E−03	4.0E−03
Photochemical oxidation	kg C2H4 eq	1.1E−03	4.1E−04	4.0E−04	4.2E−04	4.1E−04	5.1E−04	5.1E−04	5.2E−04	5.2E−04
Acidification	kg SO2 eq	6.4E−03	5.4E−03	4.4E−03	5.8E−03	4.8E−03	5.3E−03	4.6E−03	5.6E−03	4.9E−03
Eutrophication	kg PO4 eq	1.9E−03	1.6E−03	1.4E−03	1.9E−03	1.7E−03	1.8E−03	1.6E−03	2.0E−03	1.8E−03

References

1. *Environmental management—life cycle assessment- requirements and guidelines;* ISO 14044; International Organization for Standardization: Lausanne, Switzerland, 2006.
2. Lecompte, T.; Levasseur, A.; Maxime, D. Lime and hemp concrete LCA: a dynamic approach of GHG emissions and capture. In 2nd Int. Conf. Bio-based Build. Mater. 1st Conf. Ecol. Valoris. Granul. FIbrous Mater, Clermont-Ferrand, France, 21 June–23 July 2017; pp. 513–521.
3. Boutin, M.P.; Flamin, C.; Quinton, S.; Gosse, G. *Étude des caractéristiques environnementales du chanvre par l'analyse de son cycle de vie*; Direction des politiques économique et internationale: Asker New Town, France, 2006; Available online: https://agriculture.gouv.fr (accessed on 13 September 2019). (in French)
4. van Zelm, R.; Preiss, P.; van Goethem, T.; Van Dingenen, R.; Huijbregts, M. Regionalized life cycle impact assessment of air pollution on the global scale: Damage to human health and vegetation. *Atmos. Environ.* **2016**, *134*, 129–137.
5. Roy, P.-O.; Azevedo, L.B.; Margni, M.; van Zelm, R.; Deschênes, L.; Huijbregts, M.A.J. Characterization factors for terrestrial acidification at the global scale: A systematic analysis of spatial variability and uncertainty. *Sci. Total Environ.* **2014**, *500–501*, 270–276.
6. de Baan, L.; Alkemade, R.; Koellner, T. Land use impacts on biodiversity in LCA: a global approach. *Int. J. Life Cycle Assess.* **2013**, *18*, 1216–1230.
7. Huijbregts, M.A.J.; Steinmann, Z.J.N.; Elshout, P.M.F.; Stam, G.; Verones, F.; Vieira, M.; van Zelm, R. *ReCiPe A harmonized life cycle impact assessment method at midpoint and endpoint level. Report I: Characterization;* RIVM Report 2016-0104; Rijksinstituut voor Volksgezondheid en Milieu RIVM: Bilthofen, The Netherlands, Available online: https://rivm.openrepository.com (accessed on 13 September 2019) 2016.

© 2019 by the authors. Submitted for possible open access publication under the terms and conditions of the Creative Commons Attribution (CC BY) license (http://creativecommons.org/licenses/by/4.0/).
